# Frequency of subclinical thyroid dysfunction and risk factors for cardiovascular disease among women at a workplace

**DOI:** 10.1590/S1516-31802010000100005

**Published:** 2010-01-07

**Authors:** Rodrigo Diaz-Olmos, Antônio-Carlos Nogueira, Daniele Queirós Fucciolo Penalva, Paulo Andrade Lotufo, Isabela Martins Benseñor

**Affiliations:** I MD. Attending physician, Division of Internal Medicine, Hospital Universitário (HU), Universidade de São Paulo (USP), São Paulo, Brazil.; II MD, PhD. Full professor, Division of Internal Medicine, Hospital Universitário (HU), and Department of Internal Medicine, Faculdade de Medicina da Universidade de São Paulo (FMUSP), São Paulo, Brazil.; III MD, PhD. Associate professor, Division of Internal Medicine, Hospital Universitário (HU), and Department of Internal Medicine, Faculdade de Medicina da Universidade de São Paulo (FMUSP), São Paulo, Brazil.

**Keywords:** Thyroid diseases, Hypothyroidism, Hyperthyroidism, Risk factors, Cardiovascular diseases, Doenças da glândula tireóide, Hipotireoidismo, Hipertireoidismo, Fatores de risco, Doenças cardiovasculares

## Abstract

**CONTEXT AND OBJECTIVE:**

Subclinical thyroid dysfunction is very common in clinical practice and there is some evidence that it may be associated with cardiovascular disease. The aim here was to evaluate the frequencies of subclinical thyroid disease and risk factors for cardiovascular disease among women at a workplace, and to evaluate the association between subclinical thyroid disease and cardiovascular risk factors among them.

**DESIGN AND SETTING:**

Cross-sectional study on 314 women aged 40 years or over who were working at Universidade de São Paulo (USP).

**METHODS:**

All the women answered a questionnaire on sociodemographic characteristics and risk factors for cardiovascular disease and the Rose angina questionnaire. Anthropometric variables were measured and blood samples were analyzed for blood glucose, total cholesterol and fractions, high-sensitivity C-reactive protein, thyroid-stimulating hormone (TSH), free thyroxine (free-T4) and anti-thyroperoxidase antibodies (anti-TPO).

**RESULTS:**

The frequencies of subclinical hypothyroidism and hyperthyroidism were, respectively, 7.3% and 5.1%. Women with subclinical thyroid disease presented higher levels of anti-TPO than did women with normal thyroid function (P = 0.01). There were no differences in sociodemographic factors and cardiovascular risk factors according to thyroid function status, except for greater sedentarism among the women with subclinical hypothyroidism. Restricting the comparison to women with subclinical hypothyroidism (TSH > 10 mIU/l) did not change the results.

**CONCLUSION:**

In this sample of women, there was no association between poor profile of cardiovascular risk factors and presence of subclinical thyroid disease that would justify screening at the workplace.

## RESUMO

**CONTEXTO E OBJETIVO:**

A doença tireoidiana subclínica é muito frequente na prática clínica e há evidências que sugerem associação com doença cardiovascular. O objetivo foi estabelecer a frequência das doenças subclínicas da tireóide e de fatores de risco para doença cardiovascular em mulheres no local de trabalho, bem como avaliar a associação da doença tireoidiana subclínica com fatores de risco para doença cardiovascular nessas mulheres.

**TIPO DE ESTUDO E LOCAL:**

Estudo transversal em 314 mulheres com 40 ou mais anos de idade que trabalham na Universidade de São Paulo.

**MÉTODOS:**

Todas as mulheres responderam a questionário sobre características sócio-demográficas, fatores de risco para doença cardiovascular, questionário de angina de Rose, e foram realizadas medidas antropométricas e colhido sangue para dosagem de glicemia, colesterol total e frações, proteína C ultra-sensível, hormônio tireotrópico (TSH), tiroxina-livre (TS-livre) e anticorpos anti-tireoperoxidase.

**RESULTADOS:**

As frequências de hipotireoidismo subclínico e de hipertireoidismo subclínico foram respectivamente de 7,3% e 5,1%. Os níveis de anticorpos antiperoxidase foram mais elevados nas mulheres com doença subclínica da tireoide comparadas às mulheres com função tireoidiana normal (P = 0,01). Não houve nenhuma diferença estatisticamente significativa entre os fatores sociodemográficos e de risco para doença cardiovascular entre os grupos exceto pela maior presença de sedentarismo entre as mulheres com hipotireoidismo subclínico. Restringir a comparação somente às mulheres com hipotireoidismo subclínico (TSH > 10 mIU/l) não mudou os resultados.

**CONCLUSÃO:**

Nesta amostra de mulheres, não houve nenhuma associação entre um perfil inadequado dos fatores de risco para doença cardiovascular e a presença de doença subclínica da tireóide que justificasse o rastreamento no local de trabalho.

## INTRODUCTION

Subclinical thyroid dysfunction is a very common situation among middle-aged women.^1-3^ It is characterized by abnormal serum thyroid-stimulating hormone (TSH) levels with normal free thyroxine (free-T4) levels. The prevalence of subclinical hypothyroidism varies from approximately 5-6% among women of all ages to 10-17% among women over 60 years of age^1,4^ and up to 21% among women over 74 years of age.^2^ Most patients with subclinical hypothyroidism (74-87%) present serum TSH levels of between 5 and 10 mIU/l.^2,5,6^

The causes of subclinical hypothyroidism are the same as the causes of overt thyroid disease. Nevertheless, some patients with TSH levels above the upper limit of the reference range are euthyroid outliers (a term applied to the 2.5% of individuals whose TSH values are above percentile 97.5 of euthyroid distribution), and thus do not represent cases of disease. A significant proportion of such patients also have only transient thyroid axis disorders or iatrogenic subclinical hypothyroidism (undertreated overt hypothyroidism or overtreated overt hyperthyroidism).^7^ Only a small percentage of community-level individuals with subclinical hypothyroidism have real mild thyroid failure.

There is controversy about the need to aggressively search for cases of subclinical thyroid disorders, or even the need to undertake population-based screening.^1,8-13^ One possible reason for recommending such diagnostic procedures is the possibility of future complications like cardiovascular disease, neuropsychiatric symptoms or overt hypothyroidism.^1,14^

Cardiovascular disease in cases of subclinical hypothyroidism may be a consequence of cardiac dysfunction,^15-17^ atherosclerotic disease^18^ or high cholesterol.^19,20^ Although some studies have shown an association between cardiovascular disease and subclinical hypothyroidism^18,21^ or hyperthyroidism,^22^ other longitudinal studies did not show any association.^23,24^ One clear bias in some studies with positive findings was the inclusion of patients with a definitive diagnosis of thyroid disease (e.g. patients with overt hypothyroidism receiving inadequate doses of L-thyroxine, or after thyroidectomy or radioiodine therapy), and not subclinical hypothyroidism.

Regarding subclinical hyperthyroidism, the possible complications are cardiovascular disease, neuropsychiatric symptoms, reduced bone mineral density with possible fractures and overt hyperthyroidism.^1,13^ Cardiovascular disease in subclinical hyperthyroidism can be caused by cardiac dysfunction^15-17^ or atrial fibrillation.^25^

Even considering those evidences, a recent systematic review on subclinical thyroid dysfunction confirmed that most scientific/medical societies do not recommend screening for thyroid disease among general populations.^26^

Compared with men, Brazilian women present a high burden of cardiovascular mortality, similar to that found in Eastern European countries.^27,28^ In addition to the most common risk factors associated with cardiovascular disease, such as hypertension, smoking, diabetes or dyslipidemia, other factors can also be considered to be primordial determinants of cardiovascular disease.^29^ It is very important to identify primordial risk factors in the workplace,^30^ social inequalities,^31^ genetic polymorphisms^32^ and subclinical thyroid dysfunction, in order to be able to prevent them.

## OBJECTIVE

The present study had the aim of making a cross-sectional evaluation on the frequencies of subclinical thyroid disease and risk factors for cardiovascular disease among women of at least 40 years of age who agreed to be screened for subclinical thyroid dysfunction at the workplace, in a country with high female cardiovascular mortality. The study had also the objetive of making an evaluation on the association between subclinical thyroid disease and cardiovascular risk factors among these women.

## METHODS

### Subject characteristics

All 736 women aged 40 years or over who were working in three units at Universidade de São Paulo (USP) were invited to participate in screening for subclinical thyroid dysfunction. Informed consent was obtained from all these women. The study was also approved by the Ethics Committee of the University hospital.

### Sample size

We calculated the sample size for a cross-sectional study with a dichotomous variable (subclinical hypothyroidism: yes or no). The ingredients for the sample size calculation were an expected proportion of 0.10, total width of 0.10 (0.05 below and 0.05 above) and confidence level of 95%, thus giving a number of 138 women. We did the same for subclinical hyperthyroidism (yes or no) using an expected proportion of 0.10, total width of 0.05 (0.025 below and 0.025 above) and confidence level of 95%, thus giving a number of 291 women. Taking the expected proportion of 40% acceptance of the invitation for screening (expectation based on studies with volunteers in the university), we invited 736 women working in three different units in the University of São Paulo.^33^

### Data from questionnaire and anthropometric variables

All these women answered a specific questionnaire about sociodemographic characteristics and cardiovascular disease morbidity. Sedentary lifestyle was defined as exercising less than once a week. High blood pressure was evaluated both as a categorical variable (presence vs. absence of hypertension; presence of hypertension was defined as self-reported based on medical information, hospital registers or current use of medication to treat hypertension) and as a continuous variable (mean systolic and diastolic blood pressure). Diabetes was defined as a categorical variable (presence versus absence of diabetes; presence of diabetes was defined as self-reported based on medical information, hospital registers or current use of medication to treat diabetes). Hypercholesterolemia was defined as a categorical variable (presence versus absence of hypercholesterolemia; presence of hypercholesterolemia was defined as self-reported based on medical information, hospital registers or current use of medication to treat hypercholesterolemia). Angina pectoris was evaluated by means of the Rose angina questionnaire.^34^

The anthropometric measurements were made at the hospital using standardized techniques. Body mass index (BMI) was obtained by dividing the weight in kilograms by the square of height in meters. Waist circumference was measured to the nearest one millimeter at the level of midway between the lowest rib margin and the iliac crest.

We excluded from the study all women who had had any previous diagnosis or treatment for thyroid dysfunction. Subclinical hypothyroidism was defined as a TSH level > 4.0 mIU/l and a normal free thyroxine level (0.6-1.8 ng/dl); and subclinical hyperthyroidism was defined as a TSH level < 0.45 mIU/l and a normal free thyroxine level. In our analysis of the association between high-sensitivity C-reactive protein (hsCRP) and thyroid status, we discarded all hsCRP values higher than 10 mg/l, since these values were probably related to active infection or systemic inflammatory disease.^35^

### Blood samples

Blood samples were taken after a minimum of 12 hours of fasting. Total cholesterol (TC) and triglycerides (TG) were determined by means of an enzymatic colorimetric method. High-density lipoprotein-cholesterol (HDL-C) was measured in the plasma after precipitation of the low-density lipoprotein-cholesterol (LDL-C), and fasting blood glucose was analyzed using an enzymatic method. LDL-C was calculated in accordance with the Friedewald equation. TSH concentration (reference range 0.3-4.0 mIU/l) was measured by means of a third-generation immunometric assay (Roche TSH, Basel, Swiss). Free thyroxine was determined using an enzyme immunoassay (reference range 0.6-1.8 ng/dl) (Roche Free-thyroxine, Basel, Swiss) (sensitivity and specificity close to 100%). hsCRP was measured by means of particle-enhanced immunonephelometry (Dade Behring, hsCRP, Newark, USA) (sensitivity and specificity close to 100%).

### Data analysis

For categorical variables, chi-square tests were used for comparisons when appropriate. For continuous variables, analysis of variance (ANOVA) with the Bonferroni *post hoc* evaluation test was used. The age-adjusted prevalence rate of subclinical hyperthyroidism and hypothyroidism was calculated using a standard population from the World Health Organization. P values less than 0.05 were considered statistically significant. The data were analyzed using the Statistical Package for the Social Sciences (SPSS) 14.0.

## RESULTS

Out of the 736 women working in three units of USP who were invited to participate, 314 (42.7%) accepted the invitation and were included in the study. Of these, three were excluded because of the presence of overt hyperthyroidism and 11 were excluded because of the presence of overt hypothyroidism. Between the women who agreed to participate and those who did not agree, the sociodemographic and clinical characteristics were very similar, the frequencies of subclinical hypothyroidism and hyperthyroidism were, respectively, 7.3% and 5.1%. Fifty-one women were positive for antibodies against thyroperoxidase (16.2%). The mean TSH level among the women with subclinical hypothyroidism was 12.7 (± 23.0) mIU/l, but 78.3% of these women had TSH levels < 10 mIU/l.

Table 1 shows the mean values of TSH, free-T4 and anti-thyroperoxidase antibodies (anti-TPO), along with the likely causes of subclinical thyroid disease in the sample. Almost 45% of the cases were attributed to autoimmune thyroid disease. Table 2 describes the general characteristics and the distribution of cardiovascular risk factors according to thyroid status. The mean age was 47.6 (5.3) and the median was also 47 years. Most of the women in the sample were between 40 and 50 years old (only six were older than 60 years of age). There was no difference in general characteristics among these women except for sedentary lifestyle which was more common among the women with subclinical hypothyroidism.

**Table 1.** Thyroid-stimulating hormone (TSH), free-T4 levels and the likely cause of thyroid dysfunction in all women with subclinical thyroid diseases

	Subclinical thyroid disease	
	Hypothyroidism n = 23 (100%)	Hyperthyroidism n = 16 (100%)
TSH (mU/L)*	12.7 (23.0)	0.19 (0.14)
Free-T4 ng/dl*	1.0 (0.2)	1.1 (0.3)
TPO-AB (UI/ml)*	163.4 (275.8)	104.4 (170.3)
Presence of TPO-AB > 35 UI/ml	11 (47.8)	7 (43.8)
Associated cause		
Autoimmune	11 (45.8)	7 (43.8)
Post-thyroidectomy or iodotherapy	0	0
Other causes	13 (52.2)	9 (56.3)

* mean (SD); TPO-AB = thyroperoxidase-autoantibodies.

**Table 2.** General characteristics and cardiovascular risk factors among women with subclinical or normal thyroid function

General characteristics and cardiovascular risk factors	Subclinical hyperthyroidism (n = 16)	Normal thyroid function (n = 260)	Subclinical hyperthyroidism (n = 24)	P
Age (years)*	49.0 (5.0)	47.6 (5.4)	47.0 (4.4)	0.68
Number of school years*	10.0 (2.5)	10.5 (2.5)	11.4 (1.7)	0.39
Frequency of anti-thyroperoxidase antibodies (%)	43.8	9.4	47.9	< 0.005
Presence of hypertension (%)	33.3	25.0	10.5	0.55
Presence of diabetes (%)	13.3	4.1	0	0.15
History of dyslipidemia (%)	26.7	29.0	22.2	0.89
Sedentary lifestyle (%)	50.0	53.7	88.9	0.01
Angina† (%)	16.7	7.7	22.2	0.09
Smoking (%)	21.4	19.0	5.3	0.26
Body mass index (kg/m^2^)	28.0 (5.5)	26.5 (4.5)	26.2 (4.2)	0.46
Waist circumference (cm)	92.6 (19.2)	85.4 (14.0)	80.4 (12.8)	0.09
Systolic blood pressure* (mm Hg)	120.6 (13.5)	123.8 (19.7)	122.9 (17.5)	0.82
Diastolic blood pressure* (mm Hg)	81.0 (8.1)	80.3 (10.9)	81.1 (11.3)	0.92
Fasting blood glucose* (mg/dl)	112.0 (46.5)	98.3 (27.3)	90.8 (7.8)	0.08
Total cholesterol* (mg/dl)	204.9 (46.7)	198.3 (34.2)	205.9 (46.6)	0.52
High-density lipoprotein-cholesterol* (mg/dl)	56.1 (12.4)	57.1 (13.0)	57.3 (12.9)	0.95
Low-density lipoprotein-cholesterol* (mg/dl)	125.7 (34.4)	118.0 (29.7)	116.2 (44.6)	0.60
High-sensitivity C-reactive protein (hsCRP)*‡ (mg/l)	2.7 (1.7)	2.1 (2.4)	2.5 (2.1)	0.82

* mean (standard deviation);

† Angina using Rose angina questionnaire;

‡ hsCRP – ultra-sensitivity C-reactive protein ≥ 10 mg/l was excluded.

There was no difference in the distribution of cardiovascular risk factors according to thyroid function status between the groups. Restricting the comparison of subclinical hypothyroidism only to women with TSH levels higher than 10 mIU/l did not change the results except for the frequency of angina and the LDL levels. The frequency of angina in the women with subclinical hypothyroidism was lower than in the women with subclinical hyperthyroidism and the women with normal thyroid function. Although a statistically significant difference was found, it was in the inverse direction, since we had expected a higher frequency of angina in women with subclinical thyroid disease, compared with women with normal thyroid function. The same occurred for LDL-cholesterol, such that its levels were lower in individuals with subclinical hypothyroidism than in individuals with subclinical hyperthyroidism and normal thyroid function. Again, the results were in a direction that differed from what we would have expected if subclinical thyroid disease were associated with a worse profile of cardiovascular risk factors.

## DISCUSSION

The frequencies of subclinical thyroid disease and anti-thyroperoxidase antibodies were similar to those described in other countries, although slightly higher for subclinical hyperthyroidism.^1-3^ There were no differences in the distribution of cardiovascular risk factors according to thyroid function status in this sample.

Surks et al. discussed the controversy regarding reference ranges for TSH and free-T4, in 2005.^36^ Using data from NHANES (National Health and Nutrition Examination Survey), in a disease-free population, the TSH values between percentile 2.5 and percentile 97.5 ranged from 0.45-4.17 mIU/l. Based on this data, we chose our cut-offs as < 0.45 mIU/l and > 4.0 mIU/l.^3^

The Whickham survey, which was a large, good-quality population-based study with 20-year follow-up, the prevalence was 4% to 5% among women aged 18 to 44 years, and 8% to 10% among women age 45 to 74 years. For subclinical hypothyroidism, our data were very similar to the data from the Whickham study, as cited in Helfand's review in 2004.^1,36,37^ In another study, the prevalence of subclinical hyperthyroidism ranged from 0.8% to 2.5% among women over 60 years of age.^38^ The difference between those results and ours can be explained partially by the lower cutoffs used in the earlier study. If we had only classified women with TSH < 0.2 mIU/l as cases of subclinical hyperthyroidism, we would have included only 9 women, thereby showing a frequency of 2.8%, which would be closer to the previous results.

Many authors have hypothesized an association between overt hypothyroidism and cardiovascular disease, and several mechanisms may be implicated. For example, atherosclerosis may be associated with an adverse lipid profile or there may be a direct effect on myocardial or cardiovascular function.^15^ The relationship between subclinical thyroid dysfunction and cardiovascular disease could be mediated by the higher levels of serum lipids found in subclinical hypothyroidism. Some cross-sectional studies have demonstrated that the serum levels of total cholesterol and LDL-cholesterol are significantly higher in patients with subclinical hypothyroidism than in euthyroid patients.^39-42^ However, other studies have shown non-statistically significant differences between the two groups^23,40,41^ or even lower total cholesterol levels^18^ in subclinical hypothyroid patients, compared with those with normal thyroid function. There have been more than 20 intervention trials assessing the effects of L-thyroxine on the lipid profile of subjects with subclinical hypothyroidism, but most of them were uncontrolled. Many of these trials also involved iatrogenic subclinical hypothyroidism (e.g. patients taking inadequate doses of L-thyroxine) and individuals with a definitive diagnosis of thyroid disease. The studies^20,42^ that stratified TSH levels found nonsignificant effects in the patients with TSH < 10 mIU/l.

In the present cross-sectional study, there were no differences in cardiovascular risk factor profile between the women according to their thyroid function status. The sample included women who were more obese than the general population in Brazil, which is a country with an especially high risk of cardiovascular disease among women. Even for this sample of women with high levels of cardiovascular risk factors, we were unable to show any differences in cardiovascular risk according to thyroid function. Restricting the analysis to subclinical hypothyroidism among women with TSH > 10 mIU/l did not change the results.

Few studies have evaluated hsCRP, a non-classic cardiovascular risk factor among individuals with subclinical thyroid disease.^39-44^ Conflicting results have been obtained in relation to hsCRP: three studies showed some correlation,^44-46^ while another three did not show any association at all.^39,47,48^ The positive studies tended to include patients with definitive thyroid disease, as opposed to asymptomatic patients who had been found through screening. For example, the study by Christ-Crain et al.^43^ compared 63 women with subclinical hypothyroidism with 61 women with overt hypothyroidism and 40 euthyroid controls, but more than 40% of the women with subclinical hypothyroidism had been treated with radioiodine, surgery or anti-thyroid drugs for either for Graves disease or for toxic multinodular goiter. Among the remaining women with subclinical hypothyroidism, 90% had Hashimoto thyroiditis (i.e. positive for anti-thyroperoxidase antibodies). These patients are not an appropriate comparison group for community-dwelling cases of subclinical hypothyroidism. In our study, we excluded from the subclinical hypothyroidism group any women who, despite their biochemical diagnosis of subclinical hypothyroidism (elevated TSH level with free-T4 within the normal range), had already been diagnosed with thyroid disease or had been undergoing treatment with L-thyroxine.

The present study has several limitations. It is not a population-based sample and, out of the 736 women invited to participate, only 42.7% agreed and were included. Although there were no sociodemographic differences between the women who agreed to participate and the women who did not, some kind of bias is still possible. However, this was a sample of high-risk women, in which a possible association between subclinical thyroid disease and an inadequate profile of cardiovascular risk factors could easily be demonstrated. The results have implications for clinical practice, going against the notion of regular screening for subclinical thyroid diseases.

## CONCLUSION

In this sample of women with high cardiovascular risk, subclinical thyroid disease did not show any association with an inadequate profile of cardiovascular risk factors, except for sedentary lifestyle among the women with subclinical hyperthyroidism, compared with the other groups. The data from this study did not favor any recommendation for screening for thyroid function, as a regular practice in the workplace.
